# Supplementary material for: Speeding up all-against-all protein comparisons while maintaining sensitivity by considering subsequence-level homology
Source: PeerJ. 2014 Oct 7;2:e607. doi: 10.7717/peerj.607 (PMC4193403; doi:10.7717/peerj.607)
Supplement: Supplemental Information 1 — PDF containing Supplemental Figs. 1–11. [file peerj-02-607-s001.pdf]

Supplementary materials for

Speeding up all-against-all protein comparisons while maintaining sensitivity by considering subsequence-level homology

Lucas D. Wittwer, Ivana Piližota et al.

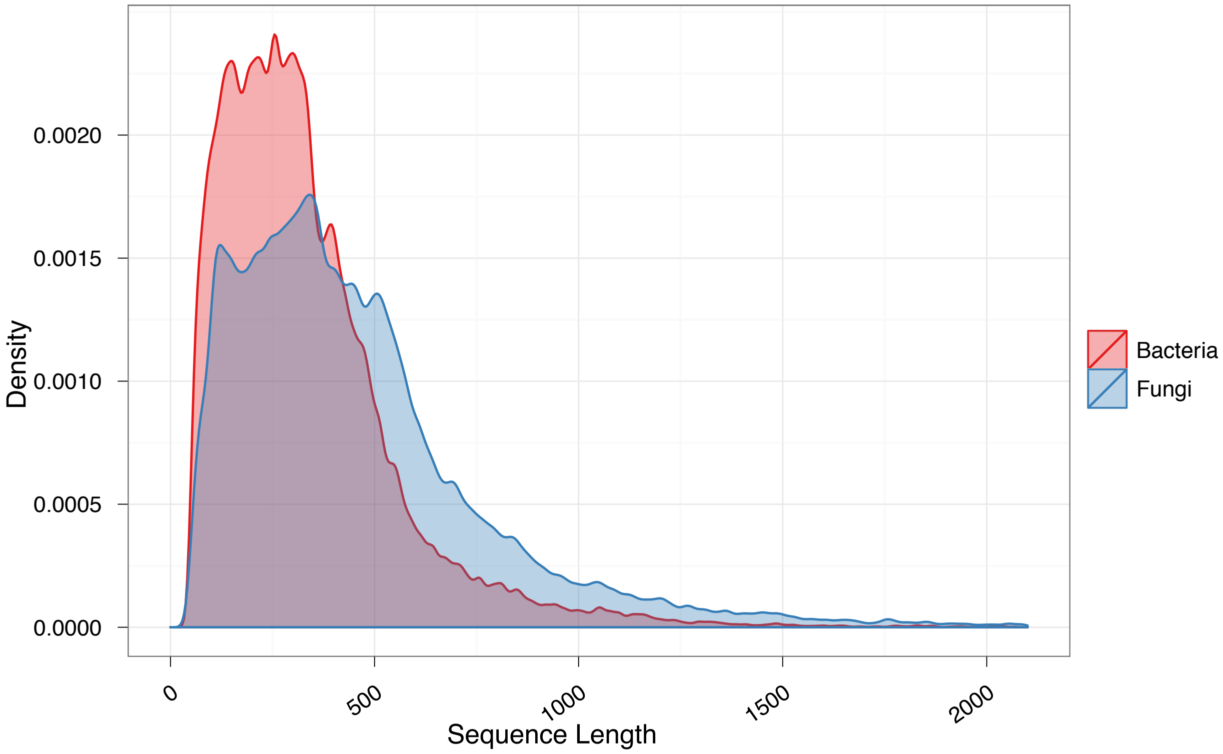

Supplementary figure 1: **Distribution of sequence length in bacteria and fungi datasets (in number of amino acids).**

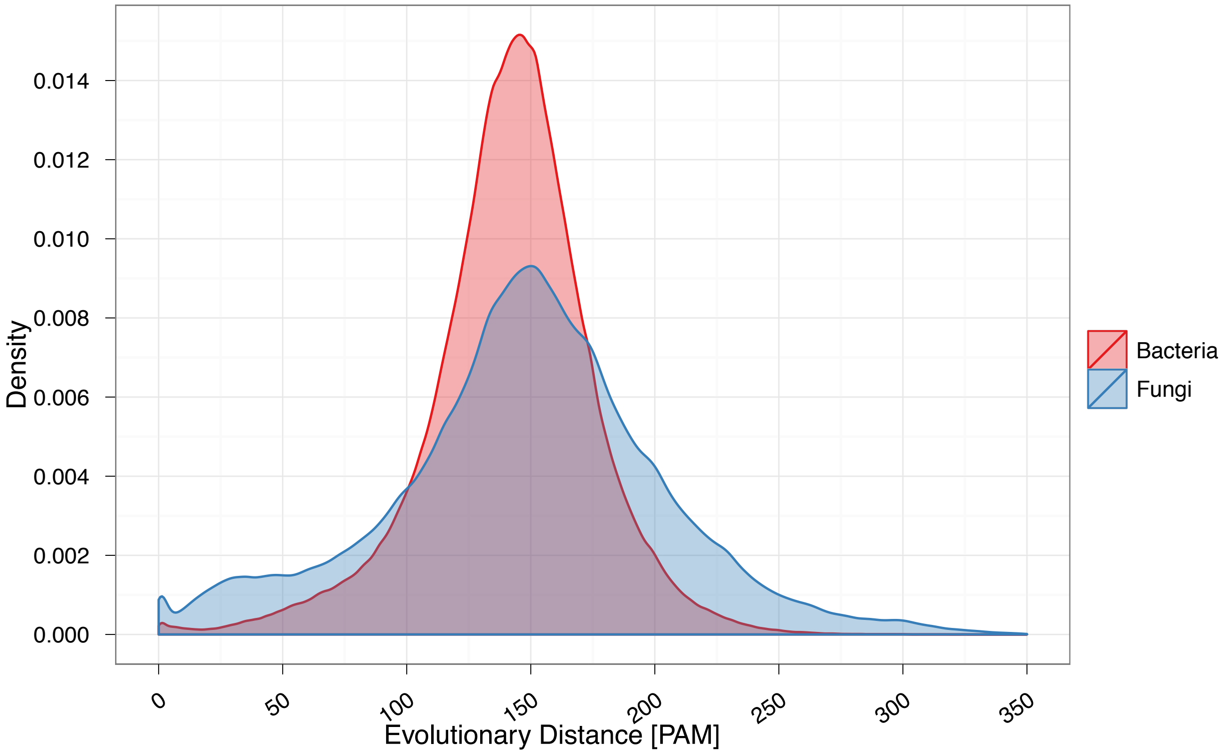

Supplementary figure 2: **Distribution of evolutionary distances among inferred homologous pairs (based on full all-against-all in bacteria and fungi datasets).**

Case studies of two missing homologs:

To illustrate the nature of missing homologs, we provide two detailed descriptions of high-scoring homologous pairs that are missed by the new approach (1 representative, subsequence homology). In both cases, one sequence is added to a cluster with an alignment score slightly above the threshold while the other is not added due to a score just below the threshold:

1) Bacteria: Sequences CHIPD1706 and CHIPD2153 (we use OMA IDs unless stated otherwise) have an alignment score of 2238.183 (estimated PAM distance: 40.3). CHIPD1706 is member of a cluster with CHIPD533 as representative because the score 141.248 is above the threshold (137.75). CHIPD1706 however is not part of the cluster because the alignment score with the representative is 117.317 only and thus below the threshold. Supplementary figure 3 depicts a multiple sequence alignment of the these three sequences, and additionally three other cluster members. Supplementary figure 4 depicts a phylogenetic tree of the sequences and confirms that the terminal branch of CHIPD2153 (outside) is slightly longer than that of CHIPD1706 (inside).

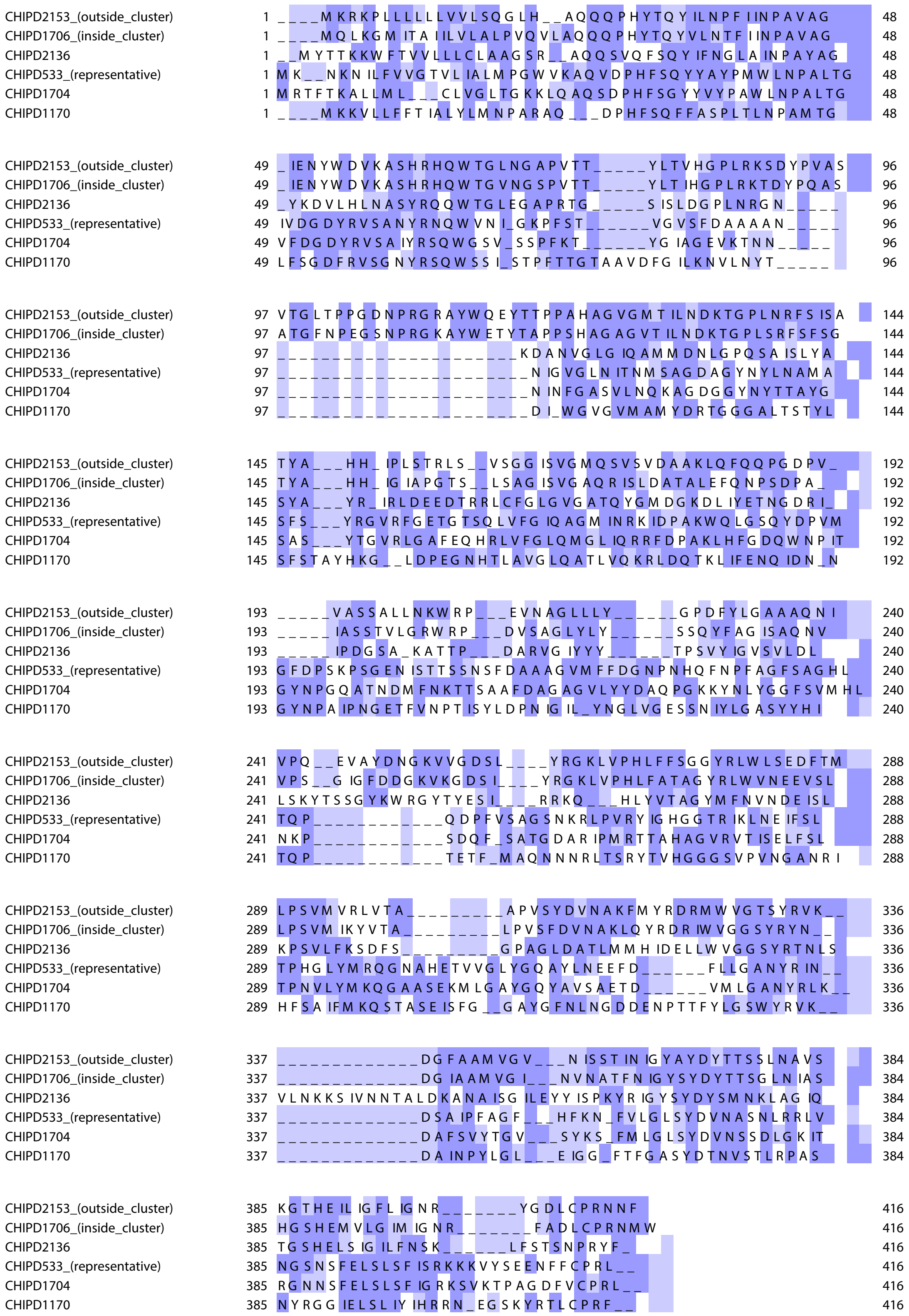

Supplementary figure 3: **Case study #1: Multiple sequence alignment of the cluster to which CHIPD2153 should be included to recover the missing pair CHIPD1706-CHIPD2153.** The corresponding tree is provided in Supplementary figure 4. Alignments drawn with JalView (Waterhouse et al. 2009 DOI:10.1093/bioinformatics/btp033)

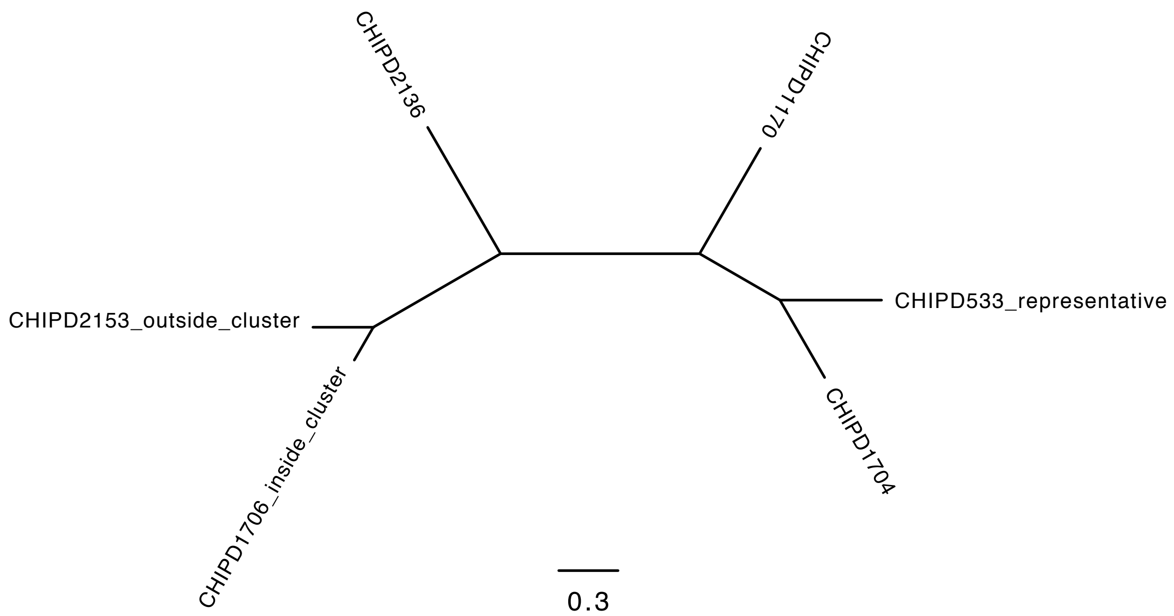

Supplementary figure 4: **Case study #1: Distance tree of the cluster and the missing sequence (CHIPD2153).** The corresponding MSA is provided in Supplementary figure 3.

2) Fungi: The homology between sequences PENCW2854 and PENCH3349 is missed despite them being nearly identical (alignment score of 5606.4 and estimated distance of 0.38 PAM units). PENCH2854 is member of two clusters—the first cluster with representative PENCH2329 (score 138.9) and the second cluster with representative PENCW2605 (score 146.6). The alignment scores of PENCH3349 with the two representatives are below the threshold (124.6 and 128.9 respectively). Supplementary figure 5 and 6 provide representative subsets of the multiple sequence alignments for the two clusters.

Furthermore, we note that both clusters are very large: each contain >1000 sequences but only a small fraction of all member pairs are significant (7.8% and 26.96%). As mentioned in the Discussion section, splitting such clusters might improve the sensitivity and runtime of the method.

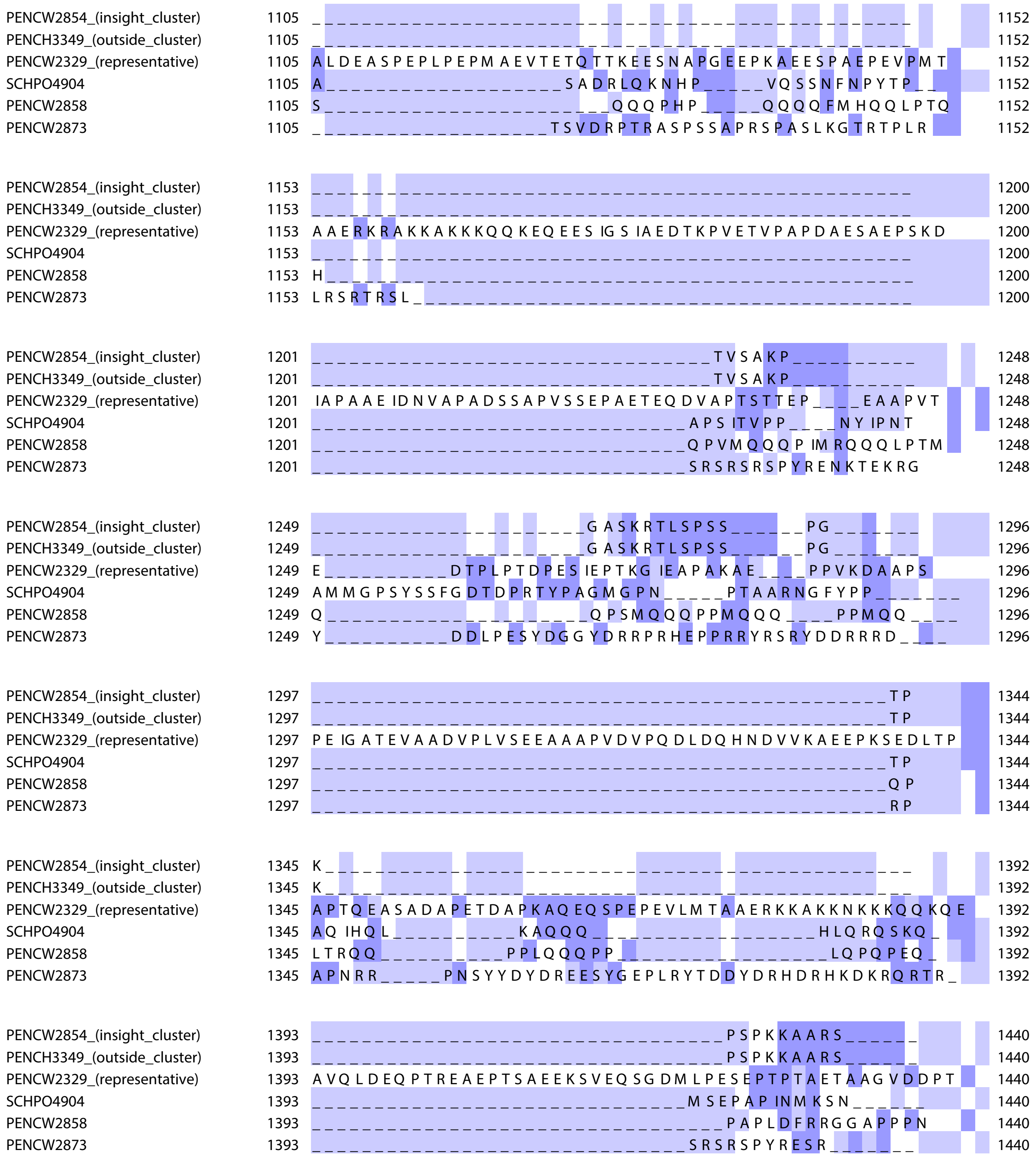

Supplementary figure 5: **Case study #2: Representative extract of the multiple sequence alignment of the first cluster to which PENCH3349 should be included to recover the missing pair PENCW2854-PENCH3349.** Alignments drawn with JalView (Waterhouse et al. 2009 DOI:10.1093/bioinformatics/btp033)

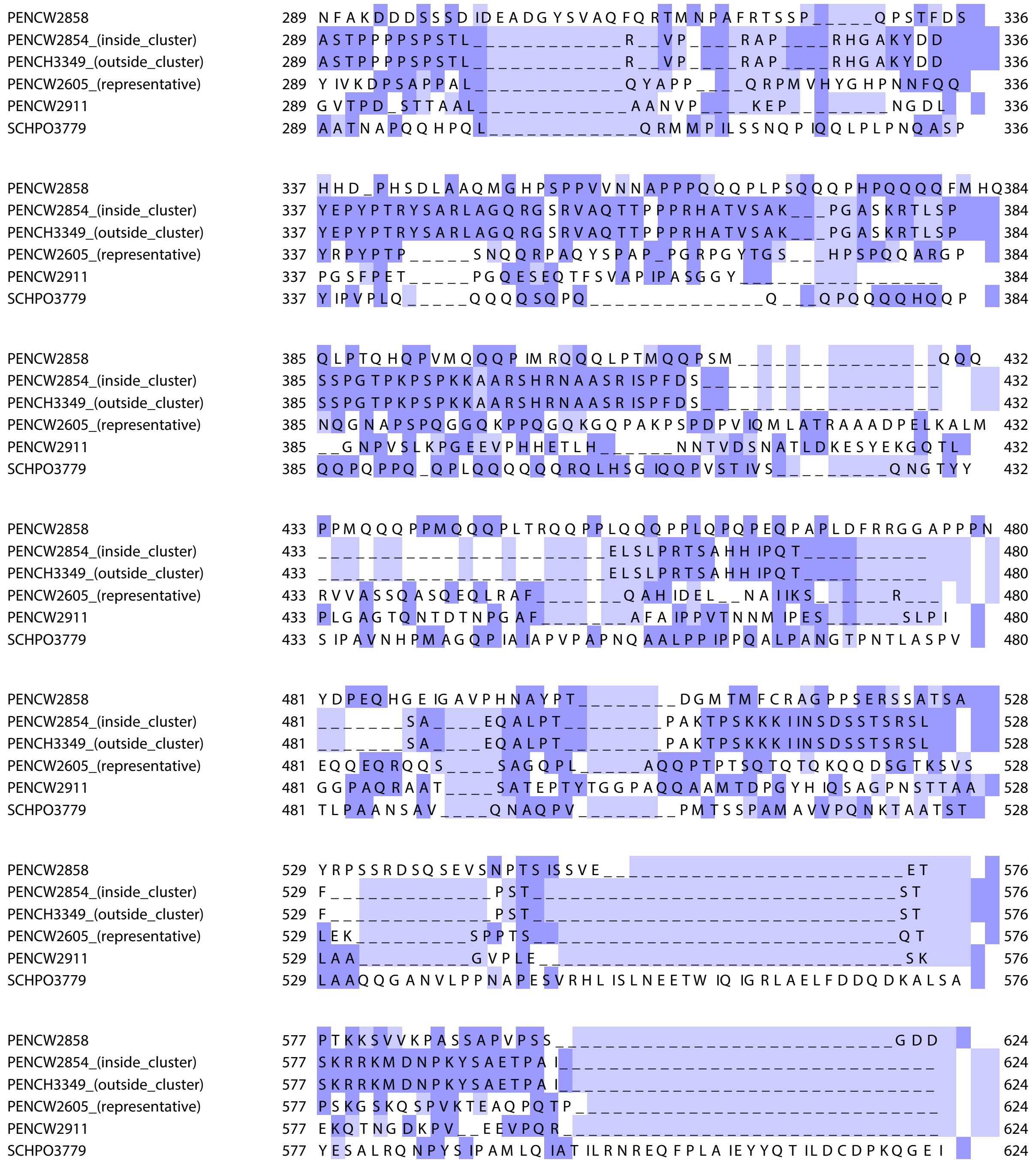

Supplementary figure 6: **Case study #2: Representative extract of the multiple sequence alignment of the second cluster to which PENCH3349 should be included to recover the missing pair PENCW2854-PENCH3349.** Alignments drawn with JalView (Waterhouse et al. 2009 DOI:10.1093/bioinformatics/btp033)

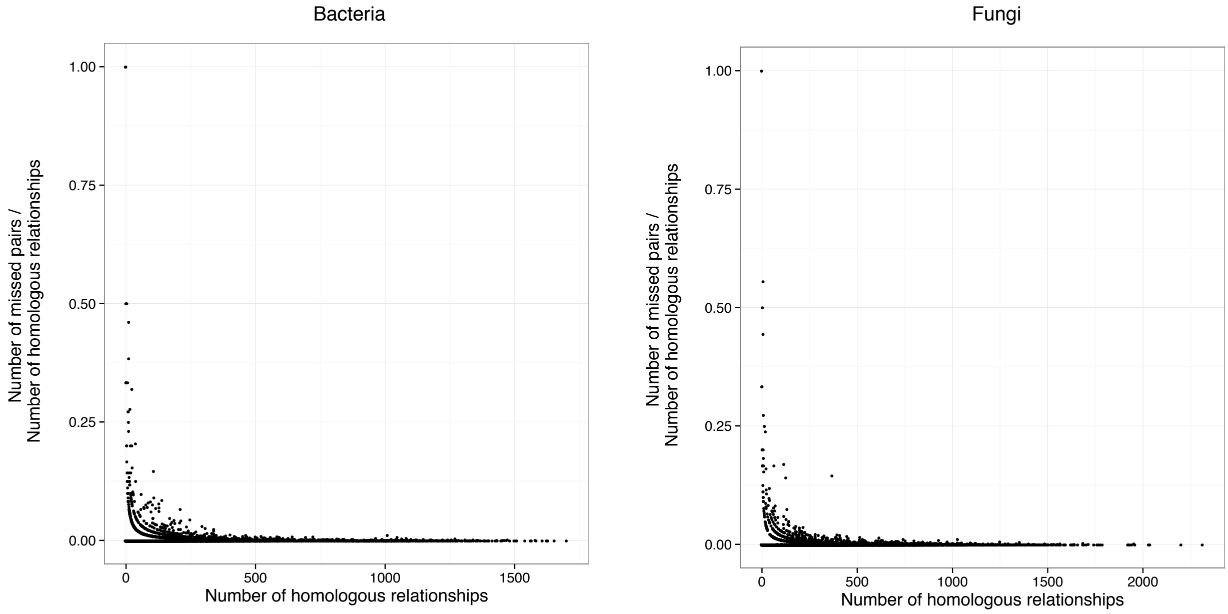

Supplementary figure 7: **Fraction of missing homologous relationships for each gene, as a function of the number of homologous relationships, for the full bacteria (14 proteomes) and fungi (12 proteomes) datasets with one representative and subsequence homology.** In large gene families (>100 members), virtually all homologous relationships are recovered compared to full all-against-all. This is also largely true for small families. The plot suggests that errors are distributed quite evenly across all types of genes.

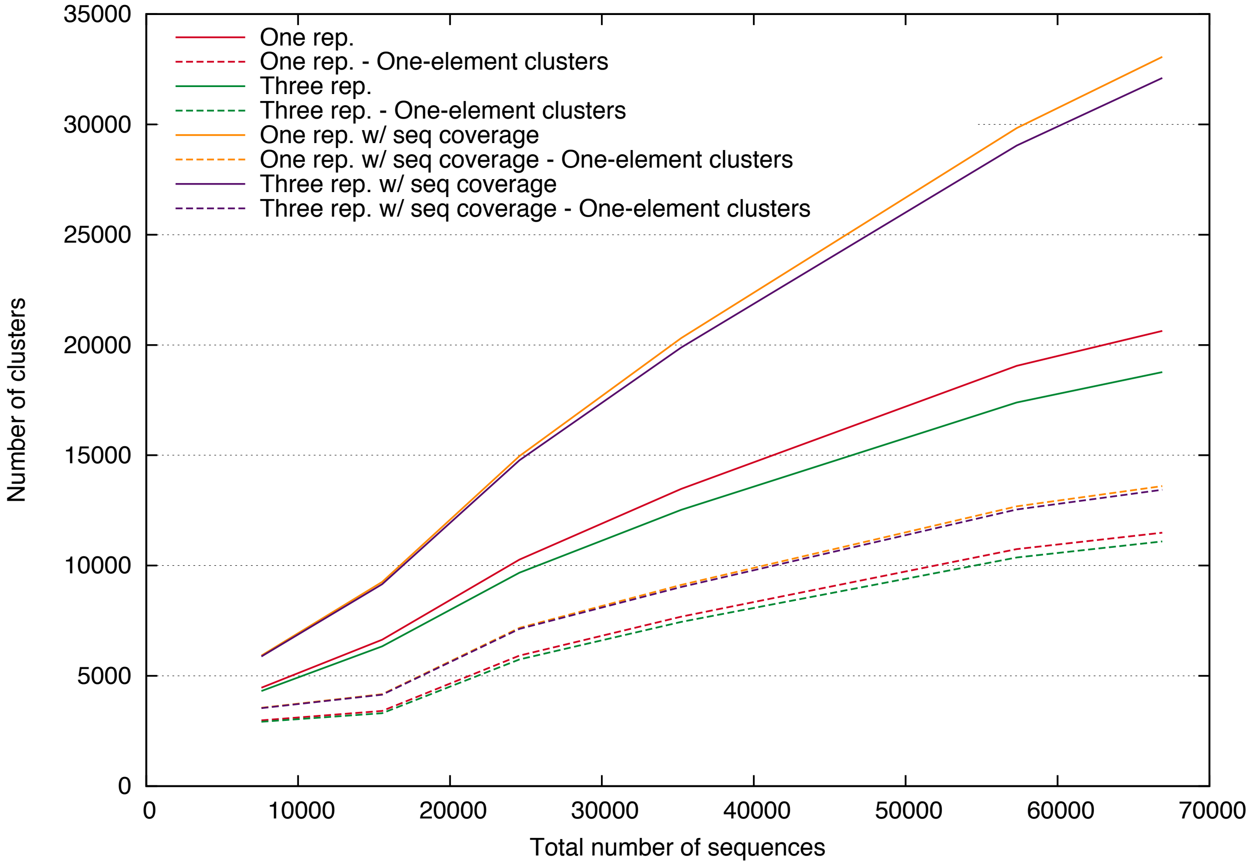

Supplementary figure 8: **Growth of number of clusters on bacteria dataset.** No tapering is observed in the growth in the number of clusters generated by the new method

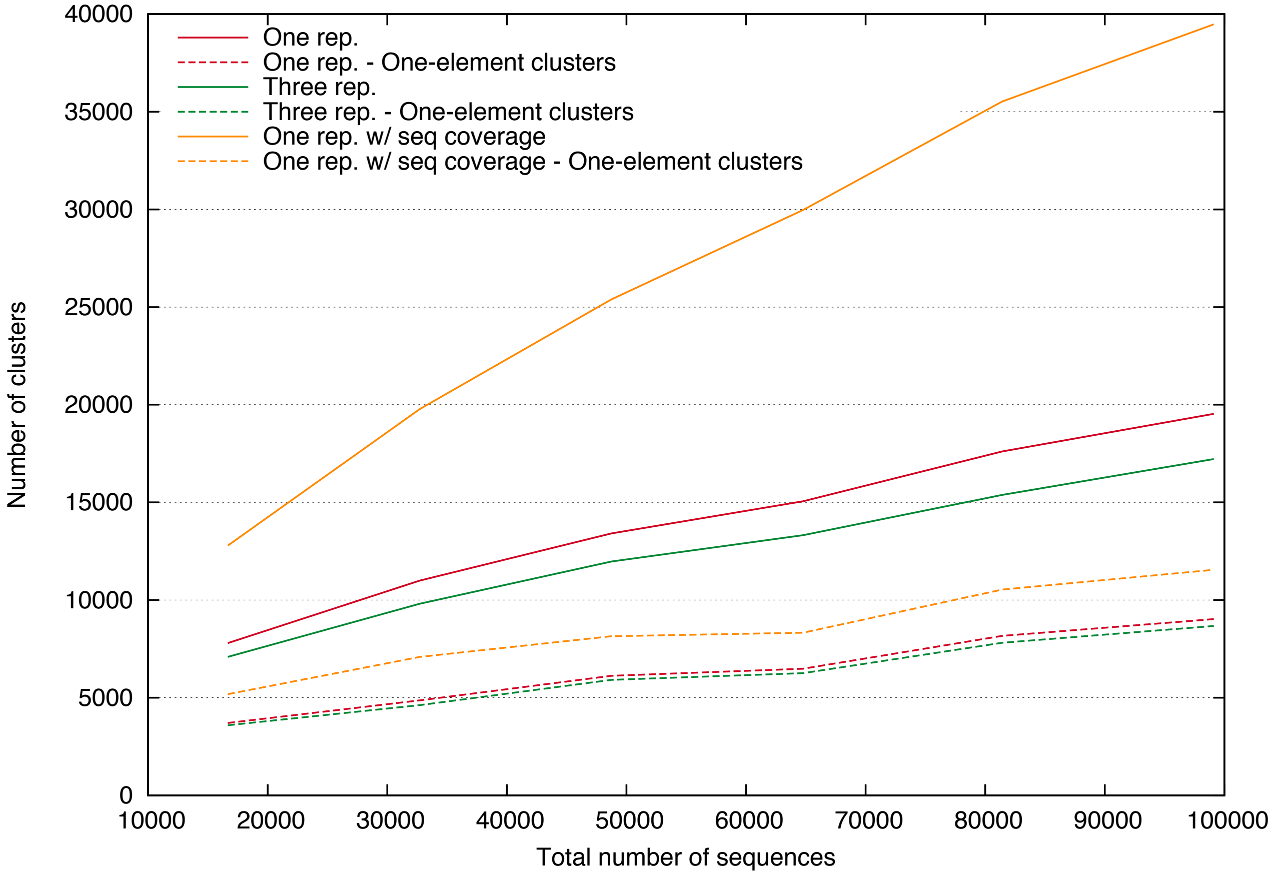

Supplementary figure 9: **Growth of number of clusters on fungi dataset.** No tapering is observed in the growth in the number of clusters generated by the new method.

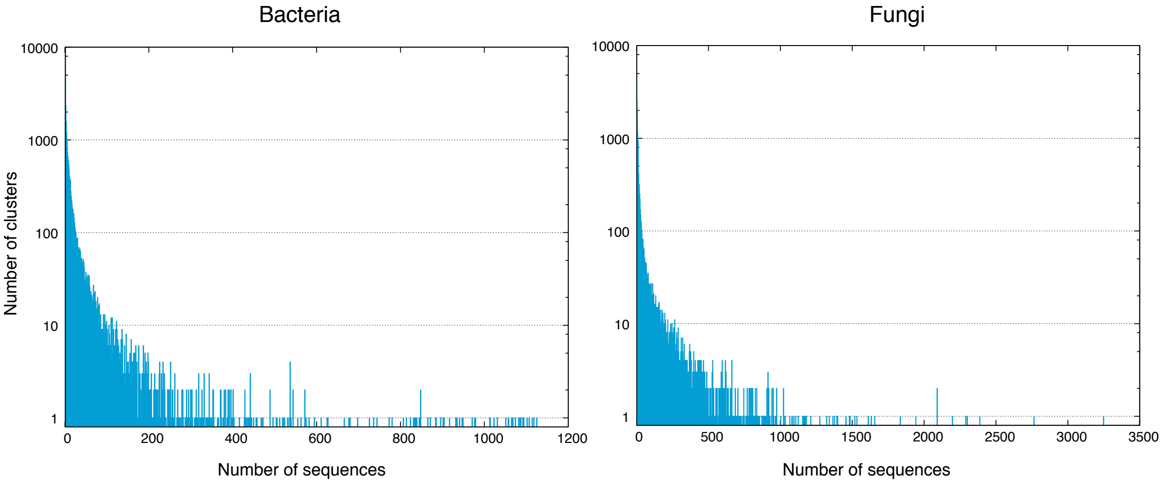

Supplementary figure 10: **Distribution of cluster size for the full bacteria (14 proteomes) and fungi (12 proteomes) datasets with one representative and subsequence homology.** The distribution is heavily skewed toward small clusters.

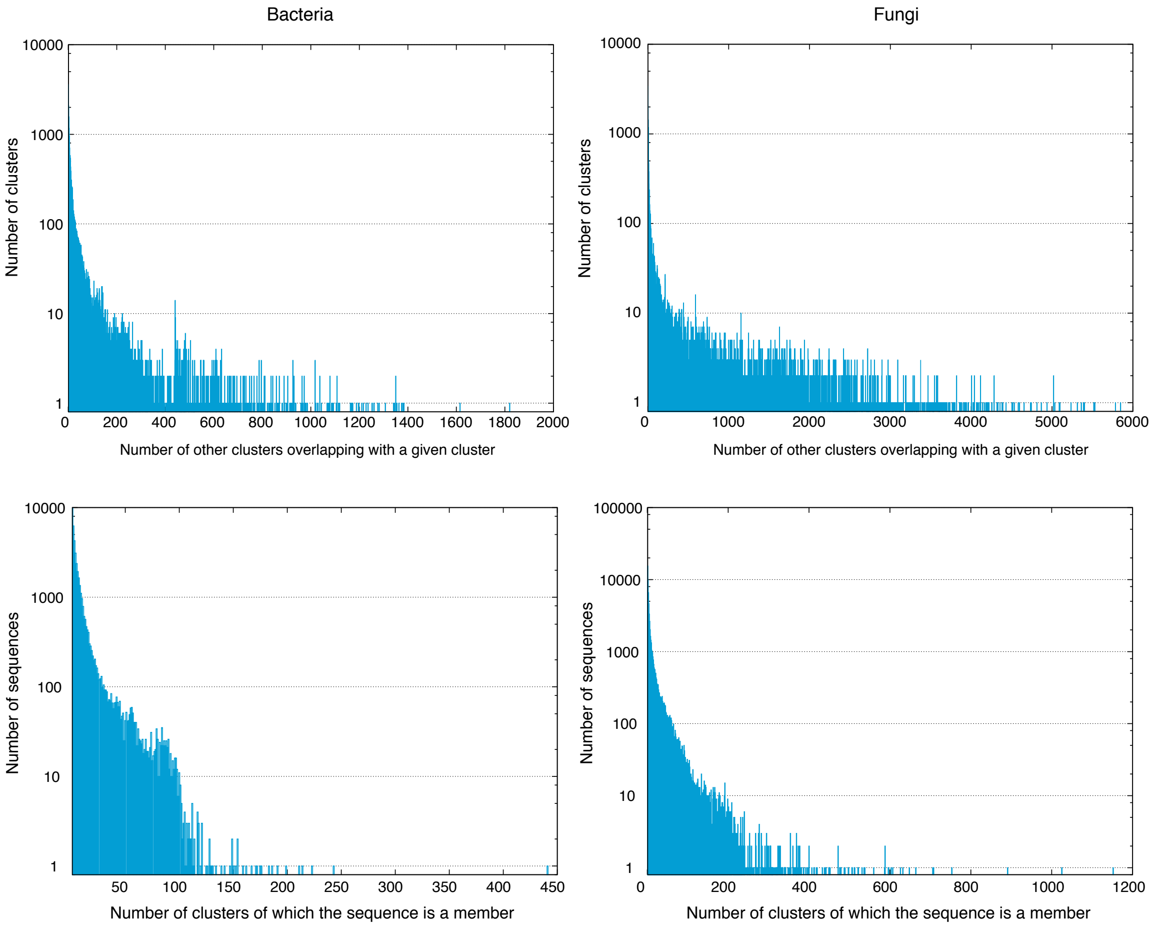

Supplementary figure 11: **Histogram of the number of clusters overlapping with each cluster (top row) and of the number of clusters in which each sequence is involved (bottom row) for the full bacteria (14 proteomes) and fungi (12 proteomes) datasets with one representative and subsequence homology.** Some clusters overlap with thousands of other clusters, which suggest potential to merge some of them (see *Discussion*).
